# Medical and Physical Intelligence

**Published:** 1800-11

**Authors:** 


### MEDICAL AND PHYSICAL

### INTELLIGENCE.

[ FOREIGN AND DOMESTIC. ]

### *Discovery of a new Salt.*

Dr. HAHNEMANN has difcovered a new fixed alkali, of which he gives the following account. It is called *Pneumalkali (Alkali pneüm)* on account of its puffing up, in a ftrong fire, to a mafs twenty times greater than before. It cryftallizes in large hexagonal prifms, ending with two oblique fides, one of which is triangular, the other pentangular. Thefe cryftals keep very well, when expofed to the moifture of the air, and neither fall into powder nor deliquefce. The *pneumalkali,* when reduced to powder, diffolves in half its weight of water, in a heat of 300° Fahrenheit, and almoft feems to liquify in its own water of chryftallization; at 65° Fahrenheit, 140 parts of it are foluble in 500 parts of water. In a great degree of cold it is extremely difficult of folution, and precipitates therefore in the cold from its neutral falts, by the addition of any other alkali. It is not foluble in fpirits of wine. By faturating a watery folution of this falt with ftrong acids, fome little ebullition is produced, with the difcharge of a fmall quantity of gas, which is not yet afcertained to be carbonic acid. The fulphat of this alkali is infoluble in fpirits of wine, and diffolves with difficulty in water. Its neutral combinations, however, with nitric, nitric, muriatic, and phosphoric acid, possess a greater degree of solubility, and particularly that with acetic acid. These neutral salts dissolve with ease in spirits of wine in great proportion, and the two last at a very cold temperature. The muriate of pneum shoots in feathery crystals; its phosphat is of a bitter taste; all neutral salts which it forms are, with the exception of the phosphat of pneum, disengaged from their acids by fire, and the pure pneumalkali is found remaining. For decomposing, however, the sulphat of pneum, it was necessary to employ the red heat. The nitrat of pneum, when liquefied in its water of crystallization by fire, discharges the acid in red fumes, and is thus decomposed by a less degree of heat. Thrown upon burning coals it causes no detonation, nor any other noise, but emits its red fumes without any illumination. A combination of this alkali with carbonic acid is very difficultly obtained, and the easiest way of performing it, is by decomposing in the cold, a solution of any neutral salt of pneumalkali by other mild alkali. The pure pneumalkali first falls to the bottom in a crystallized state, and at last the carbonat of pneum appears in the form of a light earth; but of this combination it separates already in the common temperature of our atmosphere, and this apparent earth is again changed in the space of twenty-four hours into pneumalkali. The colours of vegetables are in the same way changed by it, as by the other alkalies. It precipitates metals and earths, and even dislodges the barytes from its solution in muriatic acid, probably in a caustic state. The calcareous earth precipitated by it from the muriat of lime, dissolves in distilled water, forming on the surface of the solution, after having stood some time, a sort of cream. A solution of the pneumalkali, rubbed with sweet mercury, does not change the colour of it. Corrosive mercury precipitates with a crimson red colour, the nitrat of mercury, black; nitrated silver, white; &c. It decomposes the sal ammoniac in a heat that surpasses 100° Fahrenheit; but in the cold, even the caustic spirit of sal ammoniac, precipitates the solutions of its neutral salts. A saturated solution of this salt, unites in the cold with gross oils, when only shaken with them for a few moments, and forms a soap that is easily soluble in alkohol; and this solution may be diluted with distilled water, without becoming turbid.

These are the experiments which Dr. Hahnemann has hitherto made with this substance, from which, chemistry as well as medicine, may derive no inconsiderable advantages. It is to be sold by commission, at Chr. Gottlieb Hilsher's, of Leipsic, at one Frederic's d'or, or about seventeen shillings, for a glass containing about one ounce. Letters and money to be sent post paid. (*Crell's Chemishe Annalen, No.* 5, 1800.)

---

Prof. Mascagni of Siena, relates, in a letter to Dr. Gauturi, some observations on urinary stones and gravel. The efficacy of alkalies upon them seems to consist in neutralizing the lithic acid, and by that means forming neutral salts, which dissolve easier in water than the acid itself. The urine of persons affected with the stone, ftone, always fhews evident traces of lithic acid, whenever the fymptoms of the ftone are increafing. With this view, a folution of pure kali, was fuccefsfully employed in complaints occafioned by the ftone or gravel. But as the pure kali has the inconvenience of fometimes affecting the ftomach and bowels by its corrofivenefs, Prof. M. was curious to know whether the carbonate of potafh would have the fame effect, though he was confcious of its not acting upon ftony concretions out of the body, which are eafily diffolved in pure kali, with which the lithic acid forms a neutral falt; whereas it feems not fo nearly related with the potafh, as to difengage the carbonic acid. However, being himfelf fubject to affections of gravel, *Prof. Mascagni* began to take the carbonate of potafh, and found it very efficacious in preventing the formation of the gravel; and during the time it was ufed, the traces of lithic acid entirely difappeared, but the fymptoms increafed when it was for fome time left off. In the urine a fine faline fubftance was difcovered, that eafily difolved in warm water. By the continuance of the alkali, the urine feemed to become fuperfaturated with it, and yellow vegetable colours were changed into red by it. Thefe experiments feem to prove, that the common kali penetrates to the urinary fyftem, and prevents by its combination with the lithic acid, the formation of calculus and gravel, and it is not improbable that it likewife acts as a lithontriptic; but this is to be afcertained by farther experiments. The carbonic acid is very probably difengaged from the potafh in the way of digeftion and chylification, by which the latter is enabled to act upon the lithic acid.

Pure potafh may be given to one drachm diffolved in two pounds of water, which portion is to be taken in one day. The dofe of the carbonate of potafh is three drachms per day. Its continued ufe does not feem to have any bad effect upon the organs of digeftion. Prof. Hufeland, in whofe Practical Journal this letter is publifhed, remarks, that his own obfervations perfectly agree with the above experiments, and he always found alkalies, mineral as well as vegetable, do much good in calculous affections; and on this account the waters of *Carlsbad* in Bohemia, prove extremely efficacious againft them, as they contain a confiderable portion of mineral alkali. He frequently faw by the ufe of it, *calculous* fubftances become *sabulous*. There is a remedy celebrated in Holland, particularly under the name of *liquor lithontripticus Loosii*, which contains, according to an accurate chemical analyfis, *calx muriata*. Prof. Hufeland recommends it in the following form, ℞. *Calcis muriat. drach. j. solv. in aqu. distillat. unc. ij.* S. thirty drops to be taken four times a day, which dofe may be increafed, as far as the ftomach can bear it. (*Hufeland's Journal, Vol. ix. No.* 4.)

---

Prof. Mascagni has likewife made feveral obfervations and experiments on abforption, and the fmall capillary tubes that form the origin of the abforbent veffels, of which all the membranes of the body ſeem to be compoſed. They are of different texture, more or leſs ſolid, according to the *media* to which they are expoſed. The epidermis, for inſtance, upon which different gaſiform fluids act, conſiſts of capillary tubes of a texture more tender than thoſe which form other membranes, and by this means they are enabled to abſorb the aëriform fluidities. *(Ibid.)*

---

The late Mr. Girtanner writes in a letter to Van Mons, that he has made a number of experiments in curing venereal diſeaſes by oxygen, which diſcovery he claims to himſelf. The reſults of theſe are the following. When the diſeaſe is not inveterate, and only requires the firſt degree of oxydation, he uſes the citric acid; for the ſecond degree, the diluted oxalic acid; for the third degree, and in general againſt the moſt inveterate diſeaſes, he applies the oxyd of arſenic. There is no remedy more efficacious againſt venereal diſeaſes, diſeaſes of the liver, obſtructions of the belly, dropſies, &c. than this; but the lungs muſt not be affected, as the patient cannot then ſupport it but will expire in a ſhort time. He mixes four or five drops of a ſaturated ſolution of the white oxyd of arſenic in nitric acid, with two pints of water, to be taken in two days. This remedy has done wonders with him. There is alſo nothing more powerful againſt agues than this. If the patient begins to cough, the remedy muſt be laid aſide, a dry cough being a ſign of the body becoming over oxygenated. Should the cough continue, it can be moſtly removed by the liver of ſulphur. *(Ibid.) No.* 102.*)*

According to ſome experiments, which the ſame gentleman has made with the tranſparent rock cryſtals of Switzerland, he found that a great part of them volatiliſed like diamonds; but theſe experiments ought to be repeated. (*Annales de Chemie,* 101.)

---

Cit. Chaptal has diſcovered a new method of bleaching, whoſe peculiarity is not yet known.—The ſame gentleman has read a memoir at the Societé Philomatique, at Paris, on the art of taking out ſpots from every ſort of cloth, the contents of which we ſhall communicate in a future Number.

---

Mr. Roupen and Van Norden, two Dutch chemiſts, are at preſent occupied with new experiments on the nature of coal impregnated with different gaſes. The former has exhibited a new pyrophorus from the amber. *(Scherer's Journal of Chemiſtry.)*

---

Cit. Van Mons prepares the new ſalt, diſcovered by Chauſſier, (*Hydroſulfuré, ſulfuré de Soude: See Medical and Phyſical Journal, No. XI. Jan.* p. 82.) by expoſing a mixture of ſulphat of natron, ſulphur and charcoal, to a red heat in a covered veſſel, and by this proceſs it is obtained in conſiderable quantity. The ſulphur hydrogenates as ſoon as it gets in contact with water, and combines itſelf again as hydroſulfure with ſulphur. The hydrogen gets by this way the property of neutralifing an acid. This falt forms thick, perfectly white, and tranfparent cryftals. It does not feem to be quite as foluble in cold water as Cit. Chauffier has obferved. It detonates with the muriate of kali. Chauffier applied it with fuccefs againft herpetic exanthemata. Van Mons thinks it very ufeful againft fymptoms occafioned by the abufe of mercury. He is at prefent engaged in experiments to combine this falt with kali, ammonia, ftrontite, lime, &c. to determine the proper nature of this new preparation. (*Ibid. No.* 21.)

---

The fame chemift expofed the oxygenated pomatum to a diftillation in open fire, and obtained a great quantity of ammonia. Hence it feems evident, that the azote of the nitric acid has united itfelf with the fuet. Put upon a hot iron it got the confiftency of horn, and diffufed the fame fmell as gelatina. (*Ibid.*)

---

Dr. Woodville, who has lately returned from France, where he has been to introduce the inoculation for the Cow Pox, began the practice upon three children at Boulogne, and placed them under the care of Dr. Nowell, an Englifh phyfician, who was defired to fend vaccine matter upon lancets to Paris, as foon as the arms of thofe children produced a fufficient quantity for the purpofe. This precaution proved to be very fortunate; for, five days afterwards, when the matter of the fame pock was tried at Paris, it produced no effect whatever; and the Cow-pox matter which Dr. Thouret had received from Geneva, and which had not been longer than four or five days upon the thread, was found to be equally incapable of producing the difeafe. As Reaumur's thermometer at Paris was, about that time, frequently at 29 deg. or above 96 of Fahrenheit, it was concluded that thefe failures afforded a proof that the vaccine matter does not preferve its efficacy fo long during hot, as during temperate or cold weather. The difappointment from the above trials was not, however, of long continuance. The inoculations at Boulogne fucceeded, and from them Dr. W. was fupplied with matter, at Paris, which fully anfwered his expectations. Dr. Colon's only child was the firft perfon he inoculated at Paris; and other medical men, in order to teftify the confidence they placed in the new inoculation, followed the example; fo that Dr. W. had the fatisfaction to fee the practice extended not only among the children in different hofpitals, but alfo in private families in Paris, where, no doubt, it will foon become general. At Boulogne the Cow-pox inoculation has been continued by Doctor Nowell, who lately tranfmitted to Paris a report of the numbers to whom he had communicated the infection. With the vaccine matter which Dr. Pearfon fent to Paris, thirty children had been inoculated, of whom ten took the difeafe; from thefe ten, only five others were infected, when all further attempts to propagate the Cow-pox entirely failed, and the *matter* was loft feveral weeks before Dr. Woodville's arrival at Paris.

---

Dr.

Dr. Waterhouse, Professor of Physic at Cambridge, in New England, in a letter dated the 4th of August, 1800, to a friend at Bath, communicates the following information:

"Accept my best thanks for the phial of vaccine poison. Its activity I have experienced on my nearest connections. I have inoculated five of my own family, and roused the public attention even beyond what I had imagined. The symptoms and the appearance in the inoculated part were exactly similar to those described and pictured by Dr. Jenner. I shall try the variolous matter on them in a week or two. I consider this as a very important thing to my country, where the dread of the Small-pox is still great.

"I have ventured to give a new name to the vaccine disorder. I observe that people revolt at the term Cow-pox, I have therefore changed it to *Kine-pox*, from kine the plural of cow. As I am the only one in America who has published any thing on that new disorder, I am in hopes to establish that name instead of the former. If it should obtain, I can foresee, than the *e* will in time be changed into *d*."

The vaccine matter sent to America was taken from a patient at Bath, several months before it was employed with effect at Cambridge, in New England.

---

Dr. James Gregory, of Edinburgh, has just printed a Memorial to the patrons of the Infirmary, or Public Hospital, of that city; in which he proposes, that the surgeons of the institution shall in future be permanently attached to it; and points out many disadvantages attending the present mode, in which all the resident surgeons of the College serve in the Infirmary by rotation. In this memorial, he enters deeply into the consideration of certain differences subsisting, at present, among the surgeons of Edinburgh, and introduces many interesting anecdotes in the medical history of that celebrated school.

---

The late Dr. Kirkland has left a valuable manuscript, comprising his third volume of Medical Surgery, which is intended to be published by his son, Mr. J. Kirkland, who practises as a Surgeon at Ashby de la Zouch.

---

Mr. Noble, surgeon in Birmingham, will, in a few weeks, publish Part II. of a Treatise on the Ophthalmy, and those diseases which are induced by Inflammations of the Eyes, with methods of cure considerably different from those in common use; to which will be subjoined, an Enquiry into the Powers and Efficacy of many Applications, which are generally esteemed, and had recourse to, in different diseases of the eyes. The succeeding part will be prepared for the press as soon as possible.

---

Dr. Carson, practitioner in Midwifery, of the same town, is engaged in a small work on the Treatment of Pregnant Women, men; in which it is his intention, principally to point out the moft probable and advantageous means of preventing abortion.

---

Dr. WILLAN will fpeedily publifh Obfervations on the Difeafes of London, during the years 1796, 7, 8, 9, and part of 1800, accompanied with Meteorological Tables, and with a great variety of practical and important information.

---

Mr. JOHN BELL has made great progrefs in a work on *Military Surgery*, that has been for fome time earneftly expected from him. It will probably fill two volumes in 4to. which will contain many illuftrative engravings. Its publication will be early in the enfuing year.

---

Dr. ALEXANDER THOMSON has in the prefs, a familiar work on the cure and prevention of difeafes. He propofes to include every ufeful fact contained in Tiffot, Buchan, Wallis, Parkinfon, and other popular writers, with the improvements and recent difcoveries; and he is printing it in a fmall type and fize, that it may be fold at a moderate price.

---

